# Cytokines Explored in Saliva and Tears from Radiated Cancer Patients Correlate with Clinical Manifestations, Influencing Important Immunoregulatory Cellular Pathways

**DOI:** 10.3390/cells9092050

**Published:** 2020-09-08

**Authors:** Lara A. Aqrawi, Xiangjun Chen, Håvard Hynne, Cecilie Amdal, Sjur Reppe, Hans Christian D. Aass, Morten Rykke, Lene Hystad Hove, Alix Young, Bente Brokstad Herlofson, Kristine Løken Westgaard, Tor Paaske Utheim, Hilde Kanli Galtung, Janicke Liaaen Jensen

**Affiliations:** 1 Department of Oral Surgery and Oral Medicine, Faculty of Dentistry, University of Oslo, 0317 Oslo, Norway; LaraAdnan.Aqrawi@kristiania.no (L.A.A.); chenxiangjun1101@gmail.com (X.C.); havard.hynne@odont.uio.no (H.H.); b.b.herlofson@odont.uio.no (B.B.H.); k.l.westgaard@odont.uio.no (K.L.W.); j.c.l.jensen@odont.uio.no (J.L.J.); 2 Department of Health Sciences, Kristiania University College, 0153 Oslo, Norway; 3 Section for Head and Neck Oncology, Oslo University Hospital, 0379 Oslo, Norway; cecia@ous-hf.no; 4 Department of Medical Biochemistry, Oslo University Hospital, 0450 Oslo, Norway; sjur.reppe@medisin.uio.no (S.R.); h.c.aass@medisin.uio.no (H.C.D.A.); uxutto@ous-hf.no (T.P.U.); 5 Department of Cariology and Gerodontology, Faculty of Dentistry, University of Oslo, 0455 Oslo, Norway; morten.rykke@odont.uio.no (M.R.); l.h.hove@odont.uio.no (L.H.H.); a.r.y.vik@odont.uio.no (A.Y.); 6 Department of Otorhinolaryngology-Head and Neck Surgery Division for Head, Neck and Reconstructive Surgery, Oslo University Hospital, 0450 Oslo, Norway; 7 Department of Plastic and Reconstructive Surgery, Oslo University Hospital, 0450 Oslo, Norway; 8 Department of Oral Biology, Faculty of Dentistry, University of Oslo, 0316 Oslo, Norway; 9 The Norwegian Dry Eye Clinic, 0366 Oslo, Norway

**Keywords:** radiotherapy, head and neck cancer, cytokines, clinical manifestations, saliva, tear fluid, multiplex bead-based immunoassay, immunoregulatory signalling pathways, innate immunity, adaptive immunity

## Abstract

Although radiotherapy is a common form of treatment for head and neck cancer, it may lead to tissue damage in the salivary and lacrimal glands, possibly affecting cytokine expression in the gland fluid of treated individuals. Cytokine profiles in saliva and tear fluid of 29 radiated head and neck cancer patients and 20 controls were screened using a multiplex assay. Correlations between cytokine expression and clinical oral and ocular manifestations were examined, and cellular pathways influenced by these cytokines were assessed using the Functional Enrichment Analysis Tool. Significantly elevated cytokines identified in patient saliva were CCL21, IL-4, CX3CL1, CCL2, CXCL1 and CCL15. Many of these cytokines correlated positively with objective signs of oral dryness, and reduced saliva production in the patients. Although CCL21 and IL-4 levels were significantly lower in patient tear fluid, they correlated with subjective ocular symptoms. These increased salivary cytokines affected pro-inflammatory and apoptotic cellular pathways, including T cell signalling, several interleukin signalling pathways, TNF and TGF-β receptor signalling, and the apoptotic p53 pathway. In conclusion, the upregulated salivary cytokines identified suggest an interplay between innate and adaptive immunity, affecting immunoregulatory cellular pathways. Whether this is due to late effects of radiotherapy or tissue repair remains to be investigated.

## 1. Introduction

Head and neck cancer constitutes a group of cancers located in the pharynx, larynx, oral cavity, sino-nasal cavities and salivary glands [1]. Symptoms depend on the tumour location and include a lump and/or sore throat, and/or a mucosal ulceration that does not heal, trouble swallowing, voice change, unusual bleeding, facial swelling, numbness, and difficulty breathing [2]. It is the sixth most common cancer in the world, being responsible for 1–2% of tumour deaths worldwide [3]. When it comes to malignancies of the head and neck area, oral- and oropharyngeal cancers are the most prevalent, where squamous cell carcinoma represents more than 90% of such cases [4].

When treating head and neck cancers, radiotherapy is often employed, either alone or in combination with surgery [5,6,7,8]. In order to reduce normal tissue toxicity, the use of intensity-modulated radiotherapy has been implemented [9]. This technique maximises delivery to the targeted tissue, and minimises the dose to the surrounding healthy tissue [10]. Factors other than the radiotherapy technique used may also contribute to adjacent normal tissue damage, such as damage to the salivary and lacrimal glands [11]. These include radiation doses above 15–20 Gy, the localisation of the tumour and the volume of the targeted tissue [12].

Radiotherapy of head and neck cancer may damage salivary and lacrimal gland function, resulting in decreased saliva and tear production and symptoms such as dry mouth and dry eyes in patients. Despite decades of research, the pathogenic mechanisms associated with ocular and oral dryness are still not fully understood. Clinical tools that are implemented for measuring oral and ocular signs are often susceptible to subjective interpretation. In contrast, protein analysis has the advantage of being less prone to such subjective bias [13]. Inflammatory cytokine levels are expected to be elevated in fluids from affected glands [14]. Cytokines are intercellular signalling proteins that play a role in regulating cell growth, cellular proliferation, angiogenesis, tissue repair, and immune responses to infection, injury and inflammation [15]. Therefore, saliva and tear fluid may contain valuable biomarkers for diagnostic and therapeutic purposes [16]. Advances in the technology of multiplex bead arrays have allowed this technique to be applied in identifying proteins of low abundance in small sample volumes, while also demonstrating consistency with findings from ELISA assays [17,18]. These verifications make multiplex immunobead assays an attractive method for studying saliva and tear fluid from patients treated with radiotherapy.

Previous studies have described several salivary cytokines detected 1-3 weeks after radiotherapy in head and neck cancer patients [19,20,21,22,23]. The studies utilised both multiplex bead-based immunoassays and ELISA to identify pro- and anti-inflammatory cytokines, such as interleukin (IL)-1α, IL-1β, IL-4, IL-6, IL-8, IL-10, MCP-1, and TNF-α. These elevated cytokine levels also correlated with the radiation dose. Interestingly, in several oral and systemic conditions, including periodontal disease, Sjögren’s syndrome, and rheumatoid arthritis, levels of inflammatory cytokines are shown to increase in saliva [24]. We have previously explored cytokine levels in saliva and tear fluid from primary Sjögren’s syndrome patients in the search for disease-specific biomarkers. Our results demonstrated elevated levels of IP-10 and MIP-1a in saliva, and IL-1ra, IL-2, IL-4, IL-8, IL-12p70, IL-17A, IFN-γ, IP-10, MIP-1b, and RANTES in tear fluid [14].

To date, limited work has been published related to the concurrent investigation of cytokine profiles in saliva and tear fluid from radiated head and neck cancer patients. It is assumed that an interdisciplinary approach could increase the knowledge basis and lead to a better evaluation of late effects in these patients. Hence, in the present study, we aimed to investigate how late effects of radiotherapy might influence cytokine profiles, oral and ocular clinical outcomes, and immunoregulatory cellular pathways, in the saliva and tear fluid of radiated head and neck cancer patients, explored at least 6 months after radiation treatment. Our results demonstrate enhanced cytokine levels that suggest an interplay between innate and adaptive immune responses, affecting immunoregulatory cellular pathways, and notably, correlating with oral manifestations and ocular symptoms.

## 2. Materials and Methods

**Study participants.** This study was performed in compliance with the tenets of the Declaration of Helsinki, in the period September 2018 to March 2019. The participants included 29 patients diagnosed with head and neck cancer who had completed intensity-modulated radiotherapy treatment at least 6 months prior to recruitment, and 20 age- and sex-matched healthy individuals with no complaints of dry mouth or dry eyes. Written informed consent was obtained from all participants, and the Regional Medical Ethical Committee of South-East Norway approved the study (2018/1313).

**Figure 1:**
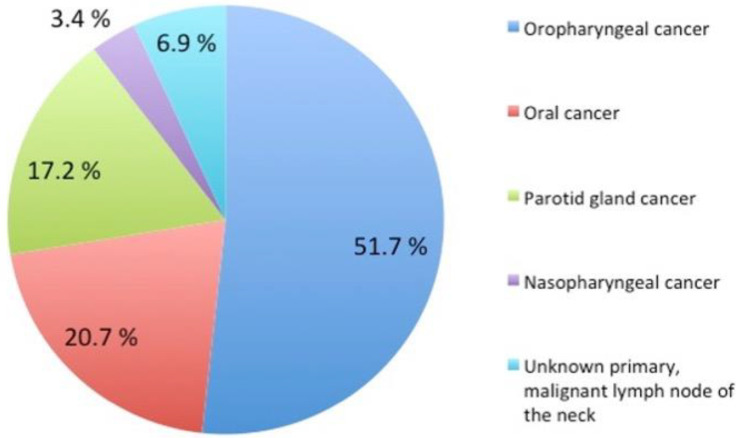
Disease location in patients with head and neck cancer (*n* = 29).

All recruited patients had undergone radiotherapy for head and neck cancer, at the Department of Oncology, Oslo University Hospital, Norway. Fourteen patients had been treated with primary radiotherapy, receiving a total dose of 68–70 Gy, and fifteen patients had received postoperative radiotherapy with a total dose of 50–66 Gy. The average radiation dose to the parotid gland was 23.1 ± 10.2 Gy (range, 1.6 to 48.5 Gy), and to the lacrimal gland 1.8 ± 4.2 Gy (range, 0.3 to 17.5 Gy). The radiotherapy was delivered as 2 Gy per fraction, administered 5–6 times per week, in accordance with standard treatment guidelines. The treatment volume varied according to tumour localisation and extension. In accordance with treatment guidelines, no shield was used to protect the lacrimal gland or the eye, since treatment volumes were distant from the eye. Furthermore, concomitant chemotherapy was given to patients under 70 years of age as part of the primary treatment for stage III-IV disease, or as part of the post-operative treatment in cases where there was marginal or perinodal infiltration. A total of twelve patients received concurrent cisplatin or cetuximab, every week for 3–6 weeks. The distribution of tumour location of the patients is shown in Figure 1, while patient demographics are presented in Table 1.

**Clinical evaluation of oral and ocular dryness.** Upon recruitment it was verified that all radiated head and neck cancer patients were currently cancer free. Individuals in the control group had no history of dry mouth/dry eye complaints, no systemic disorders or diseases with potential oral and/or ocular involvement, no previous surgery, and no use of medications that may affect lacrimal and salivary gland secretion. The oral examinations were performed at the Dry Mouth Clinic, Institute of Clinical Dentistry, Faculty of Dentistry, University of Oslo, Oslo, Norway, while the clinical ocular evaluations were performed at the Norwegian Dry Eye Clinic, Oslo, Norway, as described previously [25,26]. Participants were asked not to have anything in the mouth for at least 1 h before their oral assessment, and to avoid using any eye drops at least 2 h prior to their eye examination.

At the Dry Mouth Clinic, the Summated Xerostomia Inventory-Dutch Version (SXI-D) [27] questionnaire with a sum score (range: 5–15) was used to determine the severity of subjective feelings of xerostomia. The Clinical Oral Dryness Score (CODS) index was used to acquire an objective clinical score (range: 0–10) for oral dryness [28]. Moreover, the presence of candida growth was assessed, and unstimulated whole saliva (UWS) and chewing-stimulated whole saliva (SWS) samples were collected, as previously described [25]. Since saliva composition may be influenced by the circadian rhythm, sample collection was performed between 10:00 a.m. and 14:30 p.m. The SWS samples were aliquoted and stored at −80 °C until further cytokine analysis.

At the Norwegian Dry Eye Clinic, the severity of subjective dry eye symptoms was evaluated using the Ocular Surface Disease Index (OSDI) questionnaire. This was followed by tear quality evaluation using tear film break-up time (TFBUT) after instillation of 5 µL of 2% fluorescein sodium in each eye. Grading of corneal staining (CS) (range: 0–5) and bulbar conjunctival (ocular surface) staining (OSS) (range: 0–15) with fluorescein was recorded according to the Oxford scoring scheme [29]. Tear production was measured using Schirmer’s test, while assessment of the meibomian gland (MG) functionality and meibomian gland expressibility (ME) were assessed, as previously described [26]. Meibography images were obtained using the non-contact infrared camera system Oculus Keratograph 5 after eversion of the eyelids: meibomian gland dropout score (MGDS) in upper (UL) and lower (LL) lids was evaluated using a four-point grading scale (meiboscore) from 1 to 4 (meiboscore 1: 0–25% area loss of MG; score 2: 26–50% area loss of MG; score 3: 51–75% area loss of MG; and score 4: area loss over 75%). Tear fluid was collected using the Schirmer’s tear test strip (Haag-Streit, Essex, UK). Each Schirmer strip was transferred to an Eppendorf tube containing 500 µL of 0.1 µm filtered phosphate-buffered saline (PBS) pH 7.4 (Gibco, ThermoFisher Scientific, Oslo, Norway), and stored at −80 °C until cytokine analysis.

**Cytokine profiling of saliva and tear fluid.** Cytokine concentrations in the saliva and tear fluid, collected from all 29 patients and 20 controls, were measured using immunoassay technology (Bio-Plex xMap; Luminex Corp., Austin, TX, USA) with the commercial instrument Luminex IS 200 (Bio-Rad Laboratories, Inc., Hercules, CA, USA). Prior to analysis, Eppendorf tubes with Schirmer strips stored in PBS were thawed on ice and vortexed. Tear fluid and saliva samples were transferred into fresh tubes and diluted five folds with PBS containing BSA (final BSA concentration 0.5%). All samples were centrifuged at 10,000× *g* for 10 min at 4 °C, and 25 µL of the supernatant were then loaded onto 96-well plates.

The multiplex analysis was performed according to a previously published protocol [30]. The broad screening kit was used for the analysis (Bio-Plex Pro Human Cytokine 40-plex Assay, Cat. No. 171AK99MR2, Bio-Rad Laboratories, Inc.) and included targets against: CCL21, CXCL13, CCL27, CXCL5, CCL24, CCL26, CCL11, CX3CL1 (also known as fractalkine), CXCL6, GM-CSF, CXCL1, CXCL2, CCL1, CXCL11, IFN-γ, IL-1β, IL-2, IL-4, IL-6, IL-8, IL-10, IL-16, IP-10, CCL2 (also referred to as MCP-1), MCP-2, MCP-3, MCP-4, CCL22, MIF, CXCL9, MIP-1α, CCL15 (also called MIP-1δ), MIP-3α, MIP-3β, CCL23, CXCL16, CXCL12, CCL17, CCL25, and TNF-α.

All values obtained from the assay were in an acceptable range according to recommendations from the manufacturer (intra-percent coefficient of variation <11 and inter-percent coefficient of variation >21). Total protein concentrations in the Schirmer strip suspensions and saliva were estimated using the Pierce BCA Protein Assay Kit (Thermo Scientific, Rockford, IL, USA) and were expressed as mg/mL. The levels of cytokines were adjusted with total protein concentration and expressed as (pg of cytokine)/(mg of total protein).

**Data processing and statistical analyses.** Statistical analyses were performed using SPSS software version 25.0 (IBM Corporation, Armonk, NY, USA). Reported results were presented as means ± standard deviation. The Shapiro–Wilk test was used to assess the normality of the variables. The Mann–Whitney U test was applied to determine whether there was any statistical significance between patients and controls. Associations of cytokine levels with numerical clinical parameters and radiation dosages were determined using Spearman rank correlation. In all analyses, a *p*-value of < 0.05 was considered significant.

For functional analysis of the cytokine data, the Functional Enrichment Analysis Tool (FunRich) (http://www.funrich.org/) was applied to visualise the percentage of significantly upregulated cytokines involved in each of the affected signalling pathways. 

## 3. Results

### 3.1. Elevated Levels of Immunoregulatory Cytokines Detected in the Saliva of Radiated Head and Neck Cancer Patients Correlated with Clinical Oral Manifestations 

**Figure 2:**
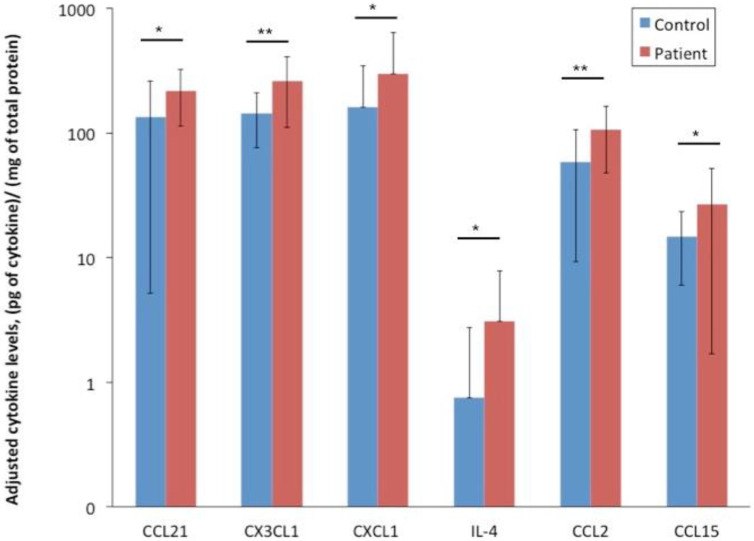
Elevated cytokine levels in the saliva of radiated head and neck cancer patients and healthy controls. Multiplex assay measurement of the cytokine levels in the patients’ saliva after radiotherapy revealed significantly higher levels of CCL21, CX3CL1, CXCL1, IL-4, CCL2, and CCL15, when compared to the controls. * represents *p* < 0.05; ** represents *p* < 0.01.

**Figure 3:**
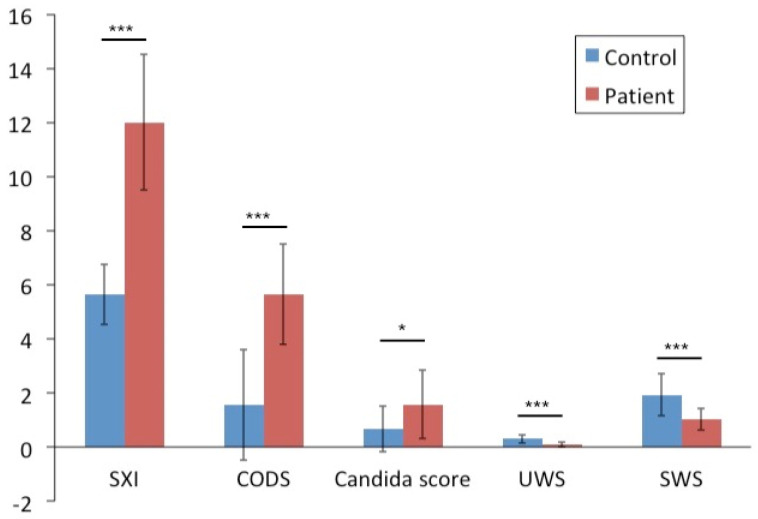
Clinical oral evaluations in patients and controls. Radiated head and neck cancer patients displayed significantly higher mean oral dryness score compared to the healthy controls in terms of both the subjective Summated Xerostomia Inventory (SXI) questionnaire and the objective Clinical Oral Dryness Score (CODS). Moreover, the patients showed lower unstimulated whole saliva (UWS) and stimulated whole saliva (SWS) production, and higher candida counts. SXI: Summated Xerostomia Inventory questionnaire score; CODS: Clinical Oral Dryness Score index; UWS: unstimulated whole saliva secretion rate (ml/min); SWS: stimulated whole saliva secretion rate (ml/min). The x-axis includes the clinical oral evaluations conducted. The y-axis illustrates the different scores attained from these examinations. * represents *p* < 0.05; *** represents *p* < 0.001.

Through the application of multiplex assay analysis, we were able to perform a broad screening of cytokines in saliva of radiated head and neck cancer patients as compared to healthy individuals. Our results revealed significantly higher levels of CCL21, CX3CL1, CXCL1, IL-4, CCL2, and CCL15 in the patient group when compared to healthy controls (Figure 2). The patients also had more subjective oral complaints, showing higher SXI-D questionnaire scores, than the healthy controls. Clinical examinations showed that the patients had a significantly higher mean objective oral dryness than controls as shown by the CODS index [28]. In addition, compared to healthy controls, the patients had lower unstimulated and stimulated whole saliva secretion rates, and higher candida counts (Figure 3). 

When viewed as a whole, the levels of upregulated cytokines correlated significantly with clinical dry mouth findings, but not with smoking status, treatment with chemotherapy, total radiation dose, nor with radiation dose administered to the parotid glands. More specifically, the level of CX3CL1 correlated with CODS (r = 0.486, *p* < 0.009) and UWS secretion rate (r = −0.420, *p* < 0.026). The level of CXCL1 was correlated with SXI (r = 0.417, *p* < 0.027), CODS (r = 0.760, *p* < 0.0001), UWS secretion rate (r = −0.433, *p* < 0.021), and SWS secretion rate (r = −0.394, *p* < 0.038). Similarly, IL-4 correlated with CODS (r = 0.595, *p* < 0.001) and UWS secretion rate (r = −0.490, *p* < 0.008), while CCL15 levels only corresponded with CODS (r = 0.511, *p* < 0.005). A more detailed representation of the relationship between summed CODS scores and the concentrations of upregulated salivary cytokines detected in each patient are presented in Table 2.

### 3.2. Cytokines Detected in the Tear Fluid of Radiated Head and Neck Cancer Patients Correlated with Clinical Ocular Symptoms

**Figure 4:**
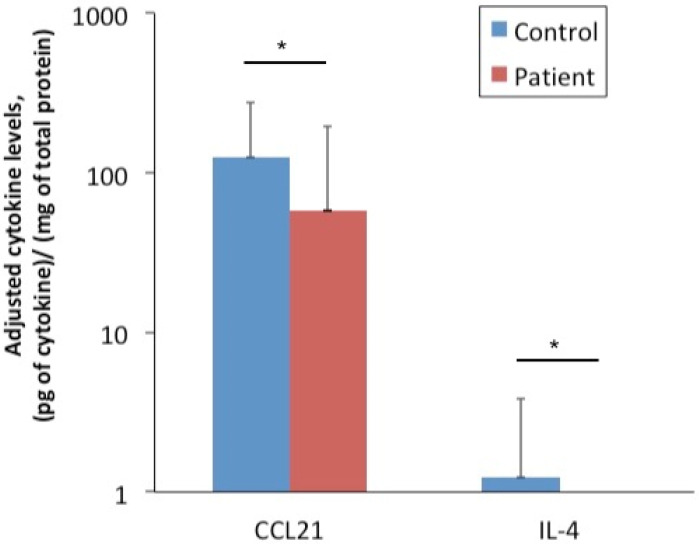
Comparison of cytokine levels in the tear fluid of radiated head and neck cancer patients and controls. Multiplex assay of the cytokine levels in tear fluid reveal significantly lower levels of both CCL21 and IL-4 in the patient group as compared to the controls. Other cytokine levels do not show statistically significant differences between patients and controls. * represents *p* < 0.05.

**Figure 5:**
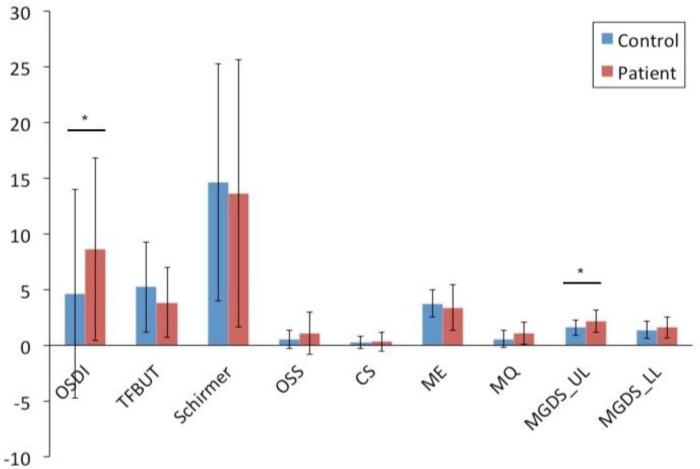
Clinical dry eye tests in patients and healthy individuals. Radiated patients show significantly more severe subjective dry eye symptoms when compared to controls, as indicated by a higher Ocular Surface Disease Index (OSDI) score, and significantly higher meibomian gland dropout score on the upper lid (MGDS_UL). Other ocular examinations do not exhibit statistically significant differences between the two groups. OSDI: Ocular Surface Disease Index questionnaire score; TFBUT: tear film breakup time; OSS: ocular surface staining; CS: corneal staining; ME: meibomian gland expressibility; MQ: meibum quality; MGDS: meibomian gland dropout score; UL: upper lid; LL: lower lid. The x-axis includes the clinical ocular evaluations conducted. The y-axis illustrates the different scores attained from these examinations. * represents *p* < 0.05.

In tear fluid, the cytokine profiling analysis showed that levels of CCL21 and IL-4 were significantly lower in the patient group than in the control group (Figure 4). Other cytokine levels did not show statistically significant differences between patients and controls. However, regarding the dry eye evaluation, the patients had more severe subjective dry eye symptoms, as shown by a higher OSDI score than the controls. The MGDS_UL was also significantly higher in the patients, whereas other ocular examinations did not demonstrate statistically significant differences between the two groups (Figure 5). When comparing the two downregulated cytokines in tear fluid with clinical dry eye tests, levels of CCL21 showed significant correlations with OSDI score (r = 0.385, *p* < 0.047) and ME (r = 0.488, *p* < 0.010), but not with other dry eye tests, smoking status, treatment with chemotherapy, nor radiation dose.

### 3.3. Cytokines Detected in Radiated Head and Neck Cancer Patients Affected Pro-Inflammatory and Apoptotic Cellular Pathways

**Figure 6:**
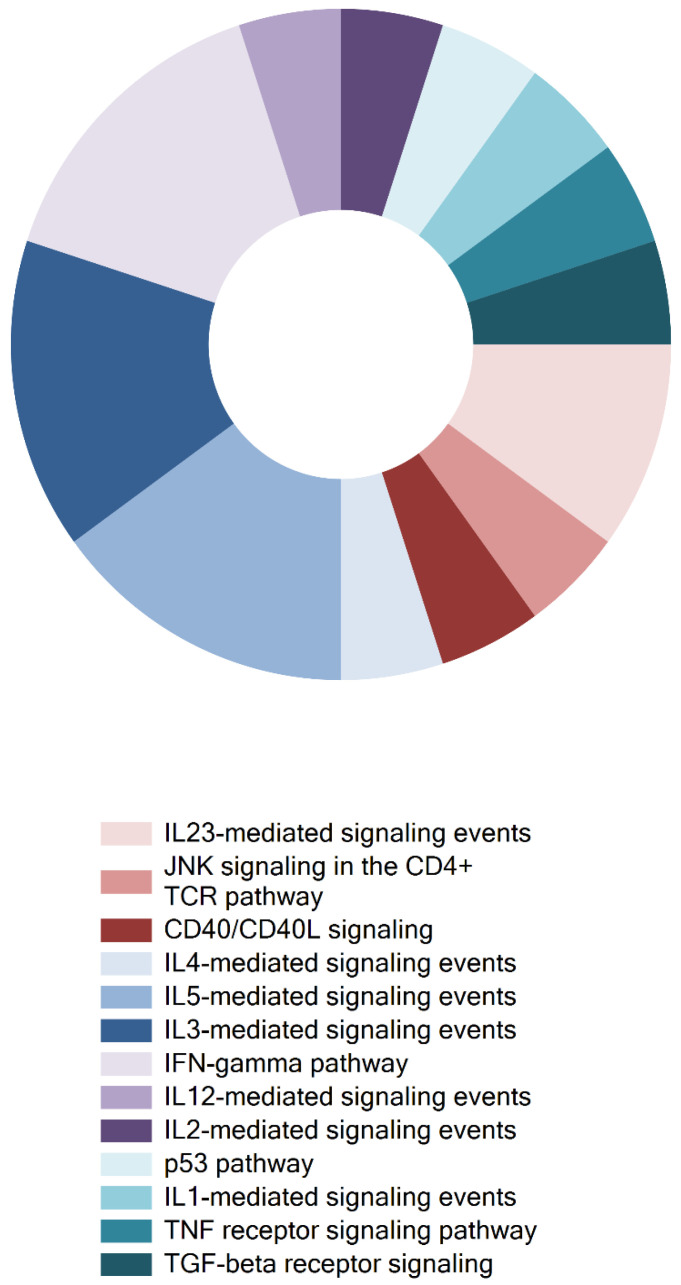
Elevated cytokine levels in saliva of radiated head and neck patients affected pro-inflammatory and apoptotic cellular pathways. The FunRich analysis of cytokines that were significantly upregulated in the saliva samples from the patients revealed which cellular pathways were influenced by these elevated cytokines, and the fractions of total upregulated cytokines that were involved in affecting these different cellular pathways. These included T cell JNK (20%), and CD40/CD40L (20%) signalling, IL-1 (20%), IL-2 (20%), IL-3 (60%), IL-4 (20%), IL-5 (60%), IL-12 (20%), and IL-23 (40%) signalling, in addition to TNF (20%) and TGF-β (20%) receptor signalling, and finally the apoptotic p53 pathway (20%).

After identifying significantly elevated cytokines in saliva, we wished to investigate which cellular pathways were affected by these particular cytokines. For such functional analysis of the cytokine data the FunRich analysis tool was applied, and the fraction of elevated cytokines influencing each of the cellular pathways detected was also estimated (presented as percentages). These pathways involved T cell JNK, and CD40/CD40L signalling (20%). Moreover, signalling pathways mediated by IL-1 (20%), IL-2 (20%), IL-3 (60%), IL-4 (20%), IL-5 (60%), IL-12 (20%), and IL-23 (40%) were also affected, in addition to TNF and TGF-β receptor signalling (20%). Finally, the apoptotic p53 pathway (20%) was also influenced by upregulated salivary cytokines (Figure 6).

## 4. Discussion

The present study marks the first time cytokine profiles were simultaneously explored in the saliva and tear fluid of patients with head and neck cancer post-radiotherapy. In spite of our patient group being rather small and heterogenous, representing subjects with primary and adjuvant radiotherapy (with and without chemotherapy), we identified significantly elevated cytokines in the saliva that have not been reported previously. This could be explained by the timing of the sample collection. In order to explore the late effects of radiotherapy, we recruited patients for cytokine analyses at least 6 months after treatment, while cytokine screening in previous reports had been conducted already 1–3 weeks after radiotherapy [19,20,21,22,23]. Therefore, the upregulated cytokines discovered in the present study could be part of a biological reaction to radiotherapy that may be associated with late effects, as indicated by the immunoregulatory and apoptotic cellular pathways identified [20]. However, their overexpression could also be due to tissue repair following radiotherapy. Radiation doses above 15–20 Gy, and treatment involving a larger volume of the targeted tissue could contribute to more severe damage to the glands [11], which may in turn trigger tissue repair mechanisms. The patients in this study received a total radiation dose of 64.5 ± 4.8 Gy (range 50 to 70), with a stronger average radiation dose directed to the saliva-producing parotid glands (23.1 ± 10.2 Gy, range 1.6 to 48.5), than to the smaller tear-producing lacrimal glands (mean 1.8 ± 4.2 Gy, range 0.3 to 17.5). In this respect, tear fluid measurements could be viewed as a control for the influence of radiotherapy, since only modest radiation doses were applied to the lacrimal glands, and our objective ocular examinations did not detect a pertinent difference between patients and controls. Notably, more prominent oral manifestations were observed in the patients, as compared to ocular findings.

Out of the six significantly elevated cytokines found when screening the saliva of these patients, CCL21 and IL-4 were significantly lower in the patients’ tear fluid than in the control participants. This may have been due to the anti-inflammatory roles of CCL21 and IL-4 in regulating disease progression. One function of CCL21 is to guide CCR7-expressing naïve T cells to T cell zones in the lymph nodes [31,32,33]. It is noteworthy that for most of the patients receiving radiotherapy to their parotid glands, the radiation field also covered the lymph nodes in the upper neck region. Hence, elevated levels of CCL21 in the saliva of the patients could be a result of tissue repair [31]. On the other hand, reduced CCL21 levels in the tear fluid of these same individuals could be a consequence of the lower radiation dose administered to their lacrimal glands, in turn leading to less tissue damage. This explanation is also supported by the fact that the objective ocular examinations did not exhibit statistically significant differences in results when comparing the patient group to the healthy controls. Furthermore, a positive correlation was only shown with the symptom-assessing ocular surface damage questionnaire (r = 0.385, *p* < 0.047), and expression ability of the meibomian glands in the eyelids (r = 0.488, *p* < 0.010). This further explains why the lower radiation dose administered to the lacrimal glands could have resulted in less severe clinical ocular manifestations.

The high level of IL-4 in saliva of the head and neck cancer patients in this study was consistent with a previous study by Citrin et al., who also found the expression levels to be dependent on the radiation dose [20]. Furthermore, IL-4 levels also correlated with clinical oral dryness scores (r = 0.595, *p* < 0.001) and unstimulated saliva secretion rates (r = −0.490, *p* < 0.008) in the patient group. Considering that IL-4-mediated cellular pathways were also affected in the FunRich analysis, this emphasises the central role of this cytokine in humoral immunity, by inducing differentiation of naïve helper T cells to Th2 cells (CD4 + Th cells), which in turn plays a central role in regulating B cell activation [20,34].

Other significantly elevated cytokines detected in the saliva of head and neck cancer patients included CX3CL1, CCL2, CXCL1 and CCL15, most of which correlated with UWS secretion rates and objective oral dryness (CODS). In particular, CXCL1 exhibited the highest concentrations that correlated strongly with high CODS values (r = 0.760, *p* < 0.0001), while other cytokines also displayed an evident association between cytokine levels in saliva and CODS, as illustrated in the colour scheme of Table 2. This further suggests a relationship between objective signs of oral dryness and cytokine production in these radiated head and neck cancer patients, a finding that exhibits relatively long after radiotherapy treatment.

Among the significantly elevated salivary cytokines identified, the CX3CL1 chemokine is known to induce chemotaxis of monocytes and cytotoxic T cells, while itself being a direct target of the tumour suppressor protein p53 [35]. Interestingly, the p53 pathway was one of the affected cellular pathways identified in the FunRich analysis, suggesting enhanced tumour suppression and apoptosis in the treated patients. Similarly, CCL2, also referred to as monocyte chemoattractant protein 1 (MCP-1), recruits monocytes, memory T cells, and dendritic cells to the sites of inflammation [36,37], as indicated in the CD40/CD40L cellular pathway identified in our study. MCP-1 has previously been detected in the saliva of head and neck cancer patients [19] soon after radiation therapy, and it remained significantly elevated in our analysis at a later time point. Meanwhile, CXCL1, expressed by macrophages, in addition to neutrophils and epithelial cells, plays a role in the processes of angiogenesis, tumorigenesis, inflammation, and wound healing [38,39]. Interestingly, it has been reported that when head and neck cancer patients with squamous cell carcinoma develop metastases, 66% of these metastases are situated in the pulmonary tissue [40]. This is also in line with the elevated levels of CCL15 in the saliva of our patient group, since this pro-inflammatory chemokine can also be expressed by macrophages in the lung tissue [41,42].

In conclusion, we have demonstrated that upregulated cytokines, particularly those identified in the saliva of radiated head and neck cancer patients, imply an interplay between innate and adaptive immune responses, affecting immunoregulatory cellular pathways, and importantly, correlating with oral manifestations and ocular symptoms. Whether these elevated cytokines are the result of late effects of treatment or the repair process remains to be explored in a larger and less heterogenous cohort in future follow-up studies.

## Figures and Tables

**Table 1:** Clinical characteristics of patients included in the study.

Patient No.	Age	Sex	Smoking Status	Type of Radiotherapy Treatment *	Total Radiation Dose (Gy)	Chemo-Therapy
1	54	Male	No	Primary	68	+
2	75	Male	No	Primary	68	−
3	63	Female	No	Primary	70	+
4	82	Female	No	Primary	68	−
5	61	Male	No	Primary	68	+
6	70	Male	No	Primary	68	+
7	69	Female	Yes	Primary	68	−
8	58	Male	No	Primary	68	+
9	67	Male	No	Primary	68	+
10	59	Male	Yes	Primary	68	−
11	53	Male	No	Primary	68	+
12	64	Male	No	Primary	68	+
13	57	Male	Yes	Primary	68	+
14	68	Male	No	Primary	68	+
15	73	Male	No	Postoperative	56	−
16	66	Female	No	Postoperative	66	−
17	65	Female	No	Postoperative	60	−
18	73	Female	No	Postoperative	66	−
19	71	Female	No	Postoperative	60	−
20	66	Female	No	Postoperative	66	−
21	51	Female	Yes	Postoperative	66	−
22	58	Male	No	Postoperative	60	-
23	41	Female	Yes	Postoperative	60	+
24	82	Male	No	Postoperative	60	−
25	51	Female	No	Postoperative	60	+
26	65	Female	No	Postoperative	66	−
27	58	Male	No	Postoperative	60	−
28	60	Female	Yes	Postoperative	50	−
29	82	Male	No	Postoperative	60	−

* All patients received radiotherapy. Some received radiation alone (primary), while others underwent excision surgery prior to radiation (postoperative).

**Table 2:** Summed CODS scores vs. concentration of upregulated salivary cytokines identified in head and neck cancer patients.

Sum CODS *	CXCL 1 **	IL-4 **	CCL 15 **	CCL21 **	CX3CL1 **	CCL2 **
8	551	12	83	418	488	163
8	570	15	40	258	242	70
8	546	8	26	162	213	52
8	424	1	36	112	172	50
8	1252	11	44	256	437	143
8	200	0	37	348	213	195
7	270	5	21	327	314	136
7	235	0	26	303	221	135
7	274	2	15	202	358	160
7	82	0	13	305	185	202
7	264	0	10	243	221	154
7	290	0	38	190	300	71
6	245	9	21	417	442	160
6	51	0	13	192	186	111
6	123	6	30	238	255	98
6	11	0	3	0	97	18
6	266	0	20	211	183	100
6	102	0	8	147	271	123
5	72	0	18	0	126	28
5	1503	11	124	389	748	211
4	152	4	24	141	331	54
4	56	0	5	133	129	47
4	228	0	12	256	507	162
3	37	0	16	173	137	47
3	188	0	14	188	142	65
3	68	0	41	167	100	24
3	158	0	6	181	125	139
2	118	0	9	164	168	49

* The Clinical Oral Dryness Score index (CODS), ranging from 0 to 10, is arranged in descending order, where the highest score has the darkest colour and the lowest score has the lightest colour. ** The concentrations of upregulated cytokines (ng cytokine per mg total protein) detected in each patient’s saliva are shown, with the highest concentrations having the darkest colour, and the lowest concentration having the lightest colour.
